# TIG welding defect detection using ResNet and Random Forest

**DOI:** 10.1371/journal.pone.0353276

**Published:** 2026-07-30

**Authors:** Vu Quang Huy, Vu Minh Thuan, Hoang Van Huong, Tran Minh The Uyen, Van-Thuc Nguyen, Pham Son Minh

**Affiliations:** 1 Faculty of Advanced Education, Ho Chi Minh City University of Technology and Engineering, HCM City, Vietnam; 2 Faculty of Mechanical Engineering, Ho Chi Minh City University of Technology and Engineering, HCM City, Vietnam; Universidad de Burgos, SPAIN

## Abstract

Orbital TIG welding is critical in precision manufacturing, where weld defects can affect structural reliability. Manual inspection, analysis, and evaluation of welding defect images are complex because defect characteristics vary in shape, position, and size, making the process time-consuming. This study proposes a method that combines ResNet50 feature extraction with Random Forest classification to improve classification accuracy. A total of 3,219 weld image samples collected from controlled orbital TIG experiments were used for model training and evaluation and divided into four classes: normal weld, lack of fusion, overheating, and uneven weld. Using five-fold cross-validation, the method achieved an overall accuracy of 98% and demonstrated improved performance compared with CNN architectures (VGG16, VGG19, ResNet18, ResNet50) and traditional machine learning approaches such as SVM and Random Forest. ResNet50 extracts hierarchical visual representations through deep residual connections, enabling automatic feature learning, while Random Forest performs the final classification with robustness against overfitting on high-dimensional features.

## 1. Introduction

Orbital Tungsten Inert Gas (TIG) welding is a specialized automated technique widely used for high-precision welding of pipes and tubular structures. In this process, a welding head moves circumferentially along a predefined path of a pipe or circular joint [1]. In practice, this method is particularly suited for industries with stringent quality requirements, including oil and gas, nuclear energy, aerospace, and medical device manufacturing. However, weld quality is strongly influenced by process parameters such as current, voltage, and welding speed [2]. Inappropriate parameter control may lead to defects including lack of fusion, overheating, porosity, and cracking, ultimately compromising structural reliability. Therefore, accurate and timely TIG welding defect detection is essential to ensure weld consistency and process stability [3–6]. For monitoring the weld pool during operation, numerous sensing approaches have been proposed. These include specular weld pool surface analysis method [7,8], ultrasonic testing [9–11], pool oscillation [6,12] and infrared sensing [13,14]. Although some systems of monitoring the welding process have been designed, recognition of weld penetration state remains limited. In addition, some other welding defects, such as misalignment, undercut and burn-through of plates are difficult to detect using conventional sensing approaches. Other studies have explored image-based approaches for weld inspection by capturing and processing weld pool images [15–17]. Radiographic and visible-spectrum images provide detailed visual information to recognize various welding states. In recent years, deep learning techniques have shown strong potential for automated weld defect classification. CNN-based models are able to learn meaningful visual patterns from complex weld images. In 2021, Lei Yang et al. [18] proposed their research weld defect location algorithm based on innovative U-net from digital X-ray images to automatically locate welding defects, including data enhancement. The proposed approach could achieve up to 88.4% accuracy on the GDXray Set. Moyun Liu et al. [19] used a lighter and faster YOLOv3 network to detect weld defects, achieving an average accuracy of 92.9%. Syed Quadir Moinuddin et al. [20] used two classification techniques including decision tree and support vector machine (SVM) to classify defects based on good welds. Their study evaluated defect classification in tube-to-tube butt joints in the flat position. Daniel Bacioiu et al. [21] developed a tungsten inert gas (TIG) welding evaluation system using a high dynamic range (HDR) camera and artificial neural networks (ANN) for image processing to automatically classify defects of TIG welding on 5083 aluminium with a four-class classification accuracy of 89%. Palma-Ramírez et al. [22] developed a deep CNN model for weld defect classification in radiographic images and reported improved classification consistency across multiple defect types. Zhang et al. [23] proposed an enhanced ResNet architecture for surface mount technology welding inspection. In industrial defect detection, Khan et al. [24] applied CNN-based models to steel surface inspection and demonstrated robustness under varying illumination and texture conditions. Similarly, Elhendawy and El-Taybany [25] introduced a machine vision-assisted welding defect detection system, emphasizing the feasibility of deploying CNN-based inspection models in real-time industrial environments. Unlike approaches relying on manually engineered features, CNN-based architectures combined with ensemble classifiers such as Random Forest can automatically learn visual representations directly from raw images, offering improved scalability and robustness for complex weld defect classification. To improve the reliable recognition of visually similar TIG weld defects, a method combining ResNet-based feature extraction with Random Forest classification is developed using optical weld images.

This paper is organized as follows. Section 2 presents the proposed methodology, including the deep feature extraction framework, ensemble classification strategy, experimental setup, dataset preparation, and training procedures. Section 3 reports and analyzes the experimental results. Finally, Section 4 concludes the study.

## 2. Methodology

**Fig 1:**
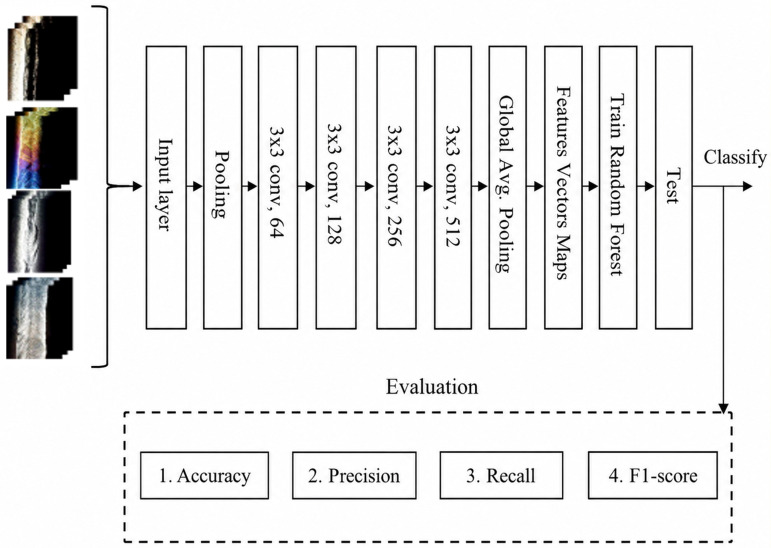
The architecture combination deep features of ResNet50 and a Random Forest classifier.

The proposed method uses deep features of ResNet50 combined with Random Forest as a classifier for the detection and grading of TIG welding defects. High-level features extracted from ResNet50 are fed to a Random Forest classifier. The classification is performed using the Random Forest (RF) classifier as shown in Fig 1 different to the traditional scheme of using the fully connected layer. With the input data being highly complex images of different states of TIG welds, the combination of ResNet and Random Forest represents a hybrid approach that leverages the strengths of both deep learning and traditional machine learning methods. ResNet excels at extracting rich and informative features from complex data, while Random Forest utilizes these features for robust classification. This integration provides several advantages. Firstly, it enhances feature extraction, allowing the Random Forest to focus on high-quality representations rather than raw data. Secondly, both ResNet and Random Forest mitigate overfitting through residual connections and bagging, respectively. Additionally, this approach is flexible: ResNet can be pretrained on large datasets and fine-tuned for specific tasks, while Random Forest can handle non-linear data effectively even with limited samples. Furthermore, Random Forest offers interpretability by analyzing feature importance, aiding in understanding the underlying data. However, training ResNet demands significant computational resources, and Random Forest’s performance can be resource-intensive when the number or depth of trees increases. The decoupled training process of ResNet and Random Forest poses optimization challenges for the entire pipeline.

### 2.1. ResNet model

A convolutional neural network is composed of three types of layers which are stacked. These are convolutional layers, pooling layers and fully-connected layers [26,27]. The convolutional layers include a series of filters, which are used for extracting deeper features from input. The pooling layers reduce the number of parameters within down-sampling along the spatial dimensionality of the input. The fully connected layer is used to combine image features and make a classification decision. Choosing a suitable CNN to classify the welding state is very important. This directly affects accuracy and practical efficiency. In welding images, the features between different welding states have a certain degree of complexity. In particular, in some cases, the impact of environmental conditions causes them to be similar. To extract high-level features, a deep network with many layers should be chosen. In this study, Residual Neural Network was applied to perform welding image classification due to its deeper network scalability.

**Fig 2:**
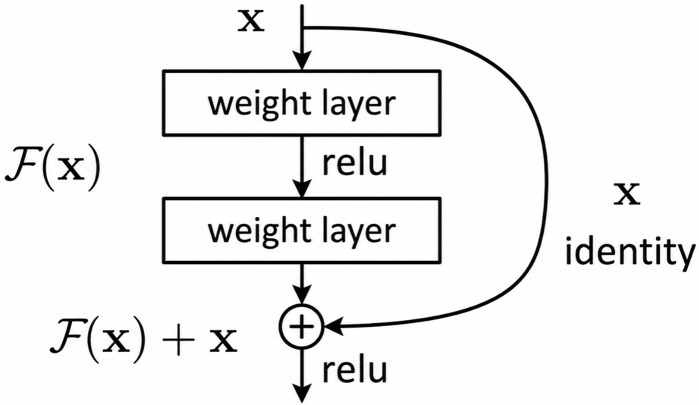
Residual block.


ResNet (Residual Network) was introduced to the public by Kaiming He [28] and ranked first in the ILSVRC and COCO 2015 competition with ImageNet Detection. CNN with many layers often suffer from the phenomenon of information degradation across layers due to many non-linear transformations stacked on top of each other, leading to a poor learning process. ResNet is designed to enable the training of extremely deep networks with thousands of layers while achieving high performance. It’s capable of solving the problems of vanishing and exposing gradients. The architecture of the residual network is made up of residual blocks. Each residual block has the operating principle as Fig 2 shown. The formula for F(x) +x can be implemented using feedforward neural networks with “shortcut connections”. Shortcut connections are connections that skip one or more layers. They only perform identity mapping and their output is added to the output of the stacked layers without adding additional parameters or complexity, preventing the derivative from being zero.

$$\begin{array}{c} H\left(x\right)=F\left(x\right)+x \end{array}$$ (1)

Where:

x: input of residual blocks

F(x): output of the layer within the residual blocks

**Fig 3:**
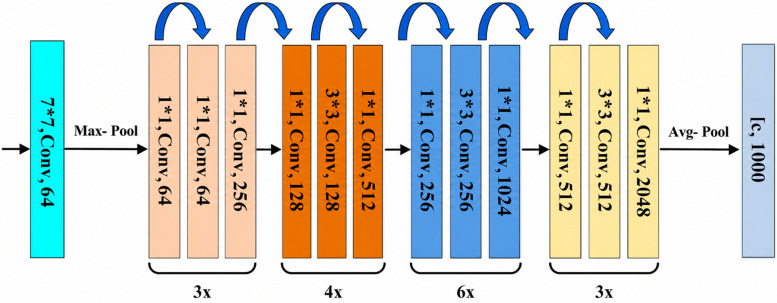
The structure of the ResNet50.

In this research, ResNet50 model with 48 convolutional layers is selected. Fig 3 shows the structure of the ResNet50. The residual shortcut connections are of two types: Black- arrow connections are used when the input and output dimensions are the same, Blue- arrow connections are used when the output dimensions are larger, achieved through zero-padding and a stride of 2. These connections allow the network to perform identity mapping even when dimensions increase.

Previous research has indicated that deep features extracted from convolutional neural networks can serve as effective representations for traditional machine learning classifiers in [29]. In industrial inspection applications, Hussain et al. [30] introduced a combined deep learning and machine learning approach to improve defect classification performance, particularly in surface defect detection for smart manufacturing systems. Therefore, ResNet50 is used as a feature extractor and Random Forest as the final classifier in this study.

### 2.2. Random Forest (RF)

Random Forest algorithm is one of the typical algorithms for ensemble learning methods [31]. Based on the principle of combining weak learners to form a strong model, it can train many decision trees in parallel by a combination of classification, bagging, and regression tree. Gini Importance equations in the Scikit-learn library used to calculate the importance of each decision tree in a Random Forest as shown in Equation (2)


$$\begin{array}{c} mi_{j}=C_{j}w_{j}-C_{\text{right}\left(j\right)}W_{\text{right}\left(j\right)}-C_{\text{left}\left(j\right)}W_{\text{left}\left(j\right)} \end{array}$$ (2)

Where:

$mi_{j}:$ importance of node j

$C_{j}:$ the impurity value of node j

$w_{j}:$ weighted samples reaching node j

$C_{\text{right}\left(j\right)}:$ the impurity value on child node from right split on node j

$C_{\text{left}\left(j\right)}:$ the impurity value on child node from left split on node j

$W_{\text{right}\left(j\right)}:$ weighted samples on child node from right split on node j

$W_{\text{left}\left(j\right)}:$ weighted samples on child node from left split on node j

The final feature importance was calculated as shown in Equation (3):


$$\begin{array}{c} {RFfi}_{i}=\frac{\sum_{j\in \text{ alltrees}}norm\;fi_{ij}}{T} \end{array}$$ (3)

Where:

${\text{norm fi}}_{ij}:$ The feature importance is normalized for feature i in tree j.

${RFfi}_{i}:$ The importance of feature i is determined from all trees in the Random Forest

T: The total of trees

Feature importance is calculated in a Random Forest as the average value of that feature importance across all trees in the forest.

### 2.3. Comparative methods

Support Vector Machine (SVM): A supervised learning algorithm used for classification and regression, which works by finding the optimal hyperplane that distinctly separates the data points into classes in a high-dimensional space [32]. VGG (Visual Geometry Group): Reputed CNN architectures (VGG16 and VGG19) characterized by their simplicity, using only 3 × 3 convolutional layers stacked on top of each other, and progressively deeper networks [33]. GoogLeNet (Inception): An innovative CNN architecture that uses an Inception module, which allows for efficient computation by using multiple kernel sizes (1 × 1, 3 × 3, 5 × 5 convolutions, and a pooling layer) within the same network block, significantly reducing the number of parameters [34].

### 2.4. Experiment setup

**Fig 4:**
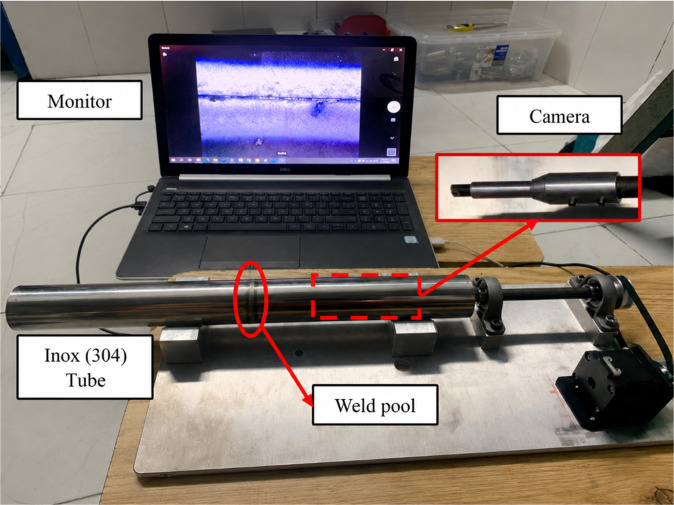
Experiment setup.

The dataset in the research is collected and provided through an experiment described as shown in Fig 4. The weld is performed on a 304 stainless steel pipe with an outer diameter of 42 mm, a wall thickness of 1.5 mm and a length of each pipe section of 200 mm. Pulse TIG welding process is used with orbital welding method with Argon gas used as shielding gas and no filler material was used. The process used DCSP with a WT20 tungsten electrode of 1.6 mm diameter. The base current was 53 A and pulse frequency ranged from 4.55 to 5.56 Hz. Welding speed was 100–105 mm/min, shielding gas flow rate was 19–22 CFH, and weld quality categories were defined in accordance with ISO 5817. A model is designed to put the camera inside the pipe to observe the weld toe. The camera used to collect data is a medical endoscopic camera with a 5.5 mm lens with a resolution of 0.3MP, a focusing distance of 15 mm, and is equipped with a light that can adjust the brightness manually. During the data collection process, the camera is mounted on an axis that can rotate 360 degrees to observe the entire weld root.

**Fig 5:**
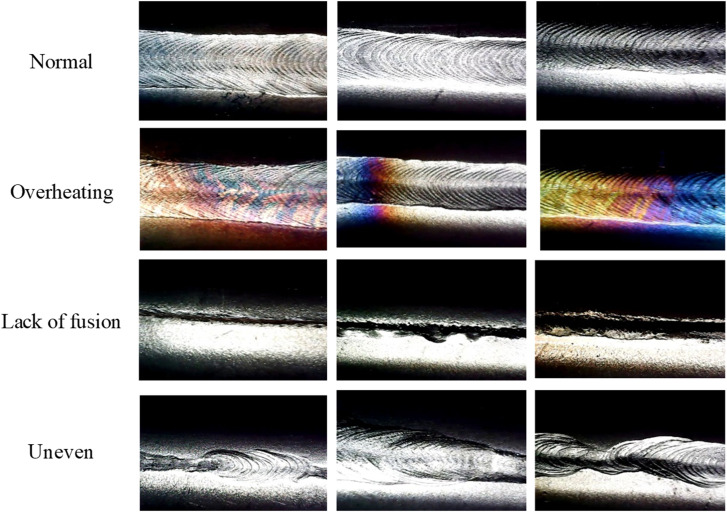
Four types of weld defect.

Experiments are designed to collect images of various welding states. In this research, four types of welding states can be obtained: lack of fusion, overheating, uneven and normal weld. Table 1 lists welding process parameters and corresponding welding states. Fig 5 depicts the different welding states collected from the experiments. Low current intensity and insufficient pulse time will cause the weld metal to not extend into the base metal to the required depth, causing incomplete penetration. Using higher amperage and appropriate pulse duration will produce a normal weld. At high currents and long pulse times, the weld will tend to be uneven and overheated.

**Table 1:** Experiment parameter.

Peak welding current(A)	Pulse time (ms)	Type of defect
115	80-90	Lack of fusion
117	100-110	Normal
120	120-130	Uneven
125	140-150	Overheat

### 2.5. Dataset

**Fig 6:**
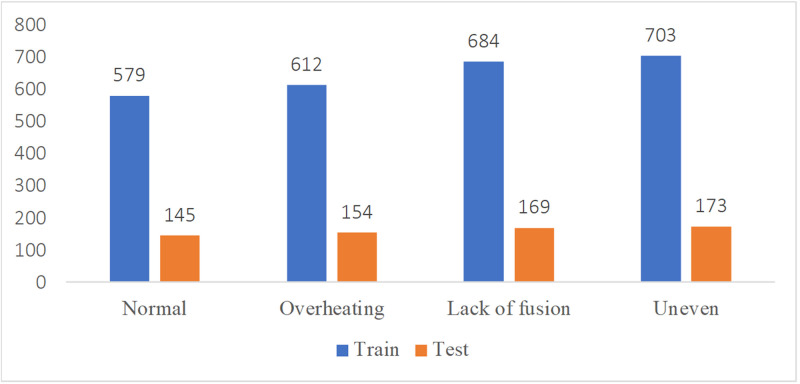
Distribution of data of dataset.

A total of 3219 samples were collected from the experiment, including 724 normal samples and 2495 defective samples. A random distribution is performed to divide the dataset into training and testing sets. There are 2578 samples in the training set accounting for 80% of the dataset and 641 samples in the test set accounting for 20% of the dataset. Fig 6 is a data distribution chart, the difference in the number of samples in each class is not too large.

For the training process to be effective, the initial dataset needs to be reprocessed. The size of the origin images in the dataset is 640x480 pixels. Images will be resized to 224x224. This helps balance between accuracy and model training time. Because the weld defects have a certain complexity, using images with a smaller size will completely lose the features of the image during the training process and lead to unexpected accuracy. Conversely, if the initial image size is too large, the model parameters will increase, which also leads to lengthening the time required for the model’s training.

**Fig 7:**
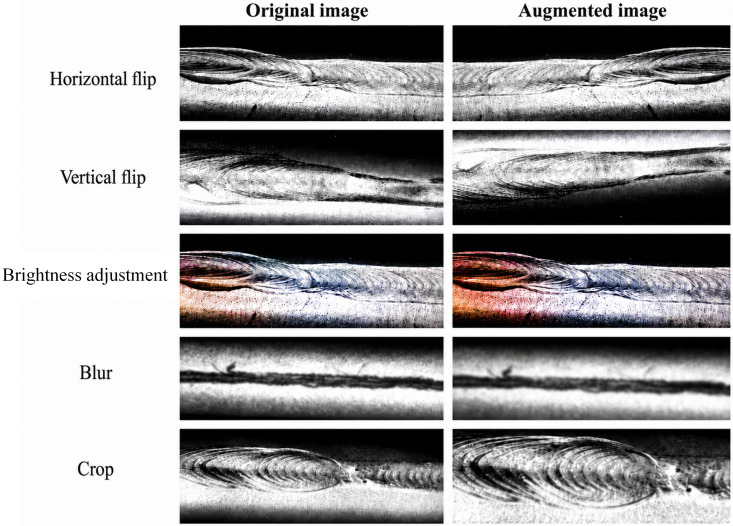
Data augmentation.

To diversify the dataset for training, data augmentation is applied. Techniques include flipping images horizontally and vertically, changing image brightness, blurring images, and cropping images. Flipping the image helps the model not depend on the position of the object. Meanwhile, changing the brightness and blurring the image will randomly create images that closely resemble the reality of what the camera captures due to shaking and focusing during movement. Additionally, performing cropping at different scales makes the model adapt to various distances and angles of the defect. Applying these methods randomly causes images to vary slightly, thereby creating a rich dataset that reduces “overfitting” and improves the performance of the trained model. As shown in Fig 7, image augmentation methods are implemented on images randomly. In machine learning, “overfitting” occurs when the resulting model is too complex to simulate the data used for training. This can lead to erroneous predictions and poor model quality on the test dataset. Then, the input image is normalized with a mean and standard deviation of [0.485, 0.456, 0.406] and [0.229, 0.224, 0.225], respectively.

These values are the standard mean and standard deviation computed from the ImageNet dataset to ensure compatibility with the pre-trained ResNet50 model. Data augmentation is performed only on the training set.

### 2.6. Evaluation method

The performance of the classifiers used in this work is evaluated by 4 parameters: (1) Accuracy, (2) Precision, (3) Recall, (4) F1-score. The metrics are used to evaluate the multi-class classifier. Four results which underlie the performance criteria of deep learning modelling techniques and evaluate their effectiveness are (true negative (TN), true positive (TP), false positive (FP), and false negative (FN)). They are often used to evaluate the effectiveness of the multi-class classification test with the total sample size in the dataset is represented by 𝑁 (class = 𝑁) [35].


$$\begin{array}{c} \text{Accuracy}=\frac{\text{1}}{N}\sum_{j=1}^{p}\frac{{TP}_{j}+{TN}_{j}}{{TP}_{j}+{FN}_{j}+{FP}_{j}+{TN}_{j}} \end{array}$$ (4)

$$\begin{array}{c} \text{Macro}-\text{\textbackslash{} Precision}=\frac{\text{1}}{N}\sum_{j=1}^{p}\frac{{TP}_{j}}{{TP}_{j}+{FP}_{j}} \end{array}$$ (5)

$$\begin{array}{c} \text{Macro}-\text{\textbackslash{} Recall}=\frac{\text{1}}{N}\sum_{j=1}^{p}\frac{{TP}_{j}}{{TP}_{j}+{FN}_{j}} \end{array}$$ (6)

$$\begin{array}{c} \text{Macro}-F1-\text{\textbackslash{} Score}=\frac{\text{1}}{N}\sum_{j=1}^{p}2\times \frac{{\text{\textbackslash{} Precision}}_{j}\times {\text{\textbackslash{} Recall}}_{j}}{{\text{\textbackslash{} Precision}}_{j}+{\text{\textbackslash{} Recall}}_{j}} \end{array}$$ (7)

## 3. Results and discussion

For training convenience, Kaggle was used in the experimentation, providing free CPU and GPU on the cloud. Pytorch was chosen to implement the deep learning framework. The datasets were first uploaded directly to the Kaggle storage and then executed using Python programming language. Five-fold cross-validation was implemented within the training set to reduce overfitting [36]. The training results of the proposed method will be presented and analyzed.

To be objective, the proposed combination method will be compared with the use of each machine learning method and the convolutional neural network alone. Random Forest and SVM [32], which are typical algorithms of machine learning, were selected on dataset. In addition, VGG [33], GoogLeNet (Inception) [34], ResNet [37] which are widely adopted CNN architectures used to train the dataset collected above. For CNN-based models, the initial learning rate was set to 0.001 and reduced by a factor of 0.1 every 5 epochs to facilitate stable convergence. A batch size of 128 samples was used, and each model was trained for 15 epochs. The CNN models were optimized using the Adam optimizer (β1 = 0.9, β2 = 0.999). The experimental results of all methods are summarized in Table 2.

**Table 2:** Comparison of different method.

Method	Accuracy	Precision	Recall	F1-score
Random Forest	0.89	0.91	0.89	0.89
SVM	0.96	0.96	0.96	0.96
VGG16	0.27	0.067	0.25	0.11
VGG19	0.27	0.067	0.25	0.11
ResNet18	0.95	0.95	0.95	0.95
ResNet50	0.95	0.95	0.95	0.95
Inception	0.97	0.97	0.97	0.97
ResNet50&Random Forest(Our study)	0.98	0.98	0.98	0.98

**Fig 8:**
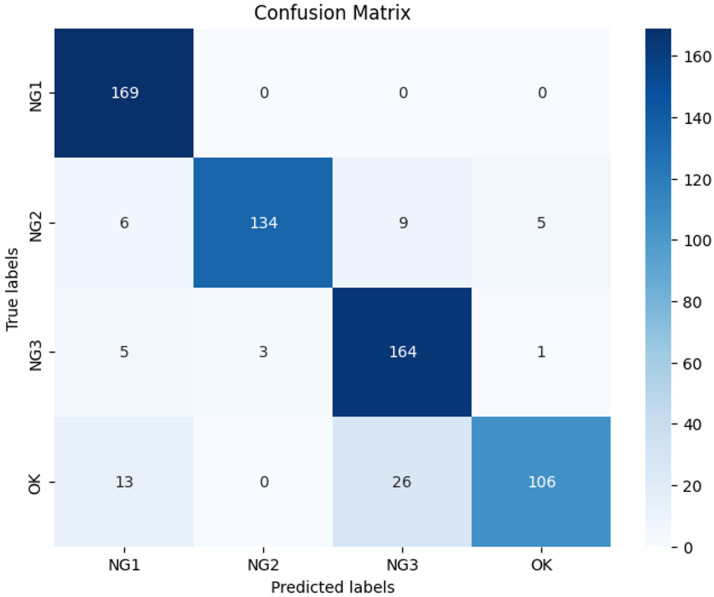
Represents confusion metric using Random Forest.

**Fig 9:**
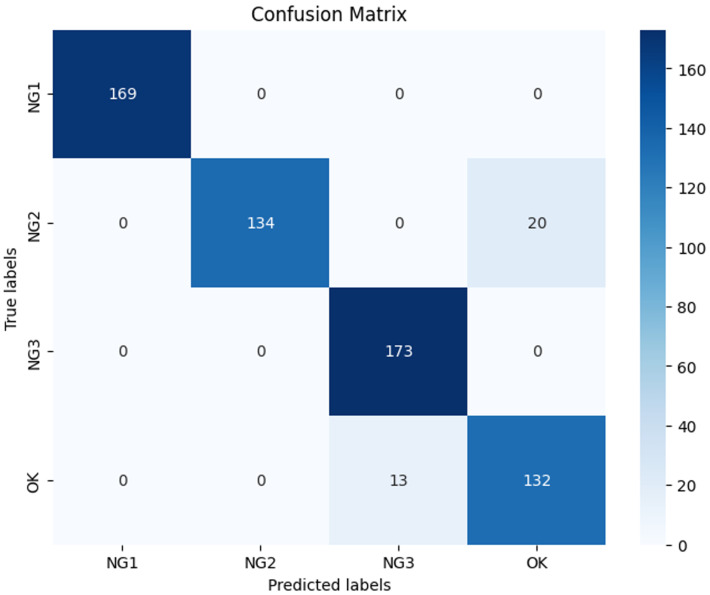
Represents confusion metric using ResNet.

**Fig 10:**
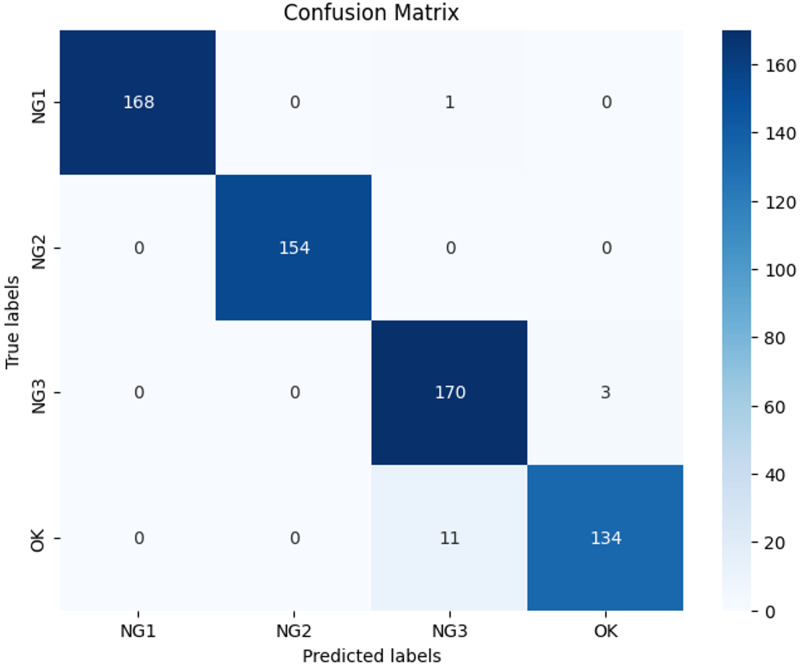
Represents confusion metric using ResNet&Random Forest.

The confusion matrix was employed to assess the performance of each individual classification method, as illustrated in Figs 8–10. The elements in each row represent the actual class of the image, while the columns represent the predicted classes. The larger the values of the diagonal elements, the greater the classification performance. This method involves four distinct classes. The heat map displays these four classes according to the labels used in our study. In Fig 8, the confusion matrix for the Random Forest method is shown, revealing that the lack of fusion class achieved the highest prediction accuracy of 100%. The prediction accuracies for the overheating, uneven, and normal classes were 87%, 94%, and 73%, respectively. A comparison in Fig 9 with the confusion matrix for ResNet shows that the lack of fusion and uneven classes were correctly classified with 100% accuracy, while the overheating and normal classes had prediction accuracies of 87% and 91%, respectively. The combination of ResNet and Random Forest in Fig 10 demonstrates that the lack of fusion and overheating classes reached 100% accuracy, while the uneven and normal classes had accuracies of 98% and 92%. Therefore, using the combination of ResNet and Random Forest, our method achieved the highest overall accuracy. Furthermore, Fig 10 shows that compared to the lack of fusion and overheating classes, the classification accuracy for normal weld and uneven weld was relatively lower, indicating potential misclassifications between these two classes. The reason for this phenomenon is that fluctuations in the welding process cause these two classes to have many similarities that even the human eye can hardly distinguish, which may lead the model to misclassify between them.

**Fig 11:**
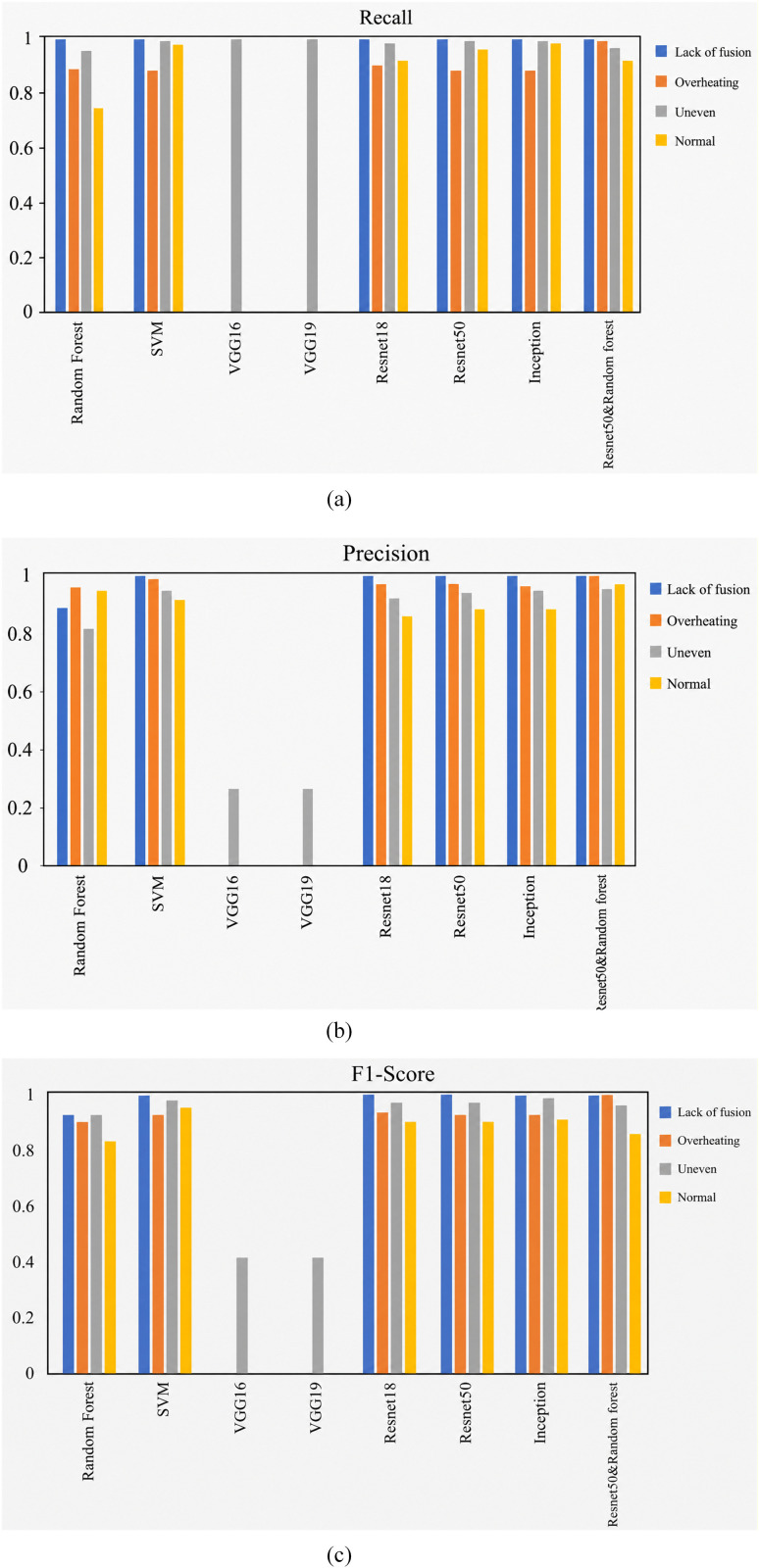
Performance classifiers: (a) Recall-score, (b) Precision-score, (c) F1-score.

Fig 11 presents the Macro-Precision, Macro-Recall, and Macro-F1 scores across different models. The proposed method achieved consistently high performance across all four weld defect categories (Normal, Lack of Fusion, Overheating, and Uneven), with overall macro metrics approaching 98%. Compared with standalone CNN architectures (ResNet and VGG) and conventional machine learning methods (Random Forest and SVM), the hybrid framework reduced performance differences among defect classes. VGG16 and VGG19 were initialized with ImageNet-pretrained weights and fine-tuned under the same training strategy as other CNN models; however, under the fixed training budget (15 epochs), their convergence was slower compared to residual architectures. The improvement was particularly evident in the Overheating and Uneven categories, where individual CNN models exhibited relatively lower recall. These results suggest that integrating deep feature extraction with ensemble-based classification improves discrimination between visually similar defect patterns.

## 4. Conclusion

In this study, a new TIG welding dataset comprising four types of weld images was introduced and used to evaluate various classification methods. The proposed approach combines ResNet50 for deep visual feature extraction and Random Forest for classification on compact feature vectors rather than raw pixel data, achieving high accuracy while maintaining computational efficiency, making it practical for identifying TIG weld quality. The model was adapted to accommodate the new TIG welding dataset, resulting in improved accuracy after training with a limited number of samples. On the same collected dataset, the above method gives better results than methods that only use traditional machine learning algorithms such as Random Forest, SVM or deep learning network architectures such as ResNet, GoogLeNet, and VGG. On the test dataset, the proposed model achieved an overall accuracy of 98%, precision 98%, recall 98%, F1-score 98%. However, accurately classifying the remaining 2% proved to be more challenging. In future work, a larger and more diverse dataset will be collected to further improve the ability of the model to classify additional weld quality categories.

## Supporting information

S1 File Data. (ZIP)
