# Supplementary material for: TIG welding defect detection using ResNet and Random Forest
Source: PLoS One. 2026 Jul 30;21(7):e0353276. doi: 10.1371/journal.pone.0353276 (PMC13422830; doi:10.1371/journal.pone.0353276)
Supplement: Attachment — Submitted filename: Response to Reviewers 2_PONE-D-25-29965_1.docx [file pone.0353276.s003.docx]

Dear Reviewer,

We would like to sincerely thank the reviewer for the careful evaluation of our manuscript and for the constructive suggestions. The comments have been extremely valuable in improving the quality, clarity, and scientific rigor of the manuscript. We have carefully revised the paper accordingly, and our detailed responses to each comment are presented below.

# Question: The title should include “welding defect detection/classification.”

**🡺 Response:**

We sincerely thank the reviewer for this helpful suggestion. The title has been revised to:

**“TIG Welding Defect Detection Using ResNet and Random Forest.”**

**Question:** The abstract needs clarification of AI methods and evaluation strategy.

**🡺 Response:**

We truly appreciate this constructive comment. The abstract has been completely revised to clearly describe:

- The applied AI methods (ResNet-50 and Random Forest),
- The evaluation strategy (five-fold cross-validation),
- The performance metrics (accuracy, precision, recall, F1-score),
- And explicitly clarify that the proposed hybrid ResNet–Random Forest model achieved superior performance compared with standalone approaches.

# Question: The references are outdated. Please include recent studies (2024–2025).

**🡺 Response:**

Thank you very much for this important recommendation. The Related Work section has been updated to include recent publications from 2024–2025, including the suggested references. This significantly strengthens the relevance and timeliness of our work.

# Question: Improvements needed in the Introduction (defect examples, structure, tense consistency, citation corrections).

**🡺 Response:**

We sincerely appreciate this detailed feedback. The Introduction has been carefully revised to:

- Include specific examples of common weld defects (porosity, cracks, lack of fusion),
- Clearly separate online and offline monitoring methods,
- Correct citation inaccuracies,
- Ensure consistent academic writing style and tense usage throughout.

# Question: Please justify the ResNet + Random Forest combination with references.

**🡺 Response:**

Thank you for this insightful suggestion. Additional references have been incorporated into the Methodology section to support the advantages of combining deep feature extraction (ResNet) with traditional machine learning classifiers (Random Forest), particularly in terms of generalization and robustness.

# Question: Please add short descriptions of the comparative models.

**🡺 Response:**

We appreciate this helpful comment. Concise descriptions of VGG, ResNet, SVM, Random Forest, and Inception models have now been included in the Methodology section for better clarity and completeness.

# Question: Is the endoscopic camera sufficient for weld defect detection?

**🡺 Response:**

We sincerely thank the reviewer for this thoughtful question. The selected endoscopic camera (640 × 480 resolution, 15 mm focus distance) provides a spatial resolution of approximately **0.03–0.05 mm per pixel**, which is sufficient to detect millimeter-scale weld defects within pipe geometry. Stable mounting and controlled illumination ensured consistent and reliable image acquisition. This justification has been added to the manuscript.

# Question: How were welding parameter ranges selected?

**🡺 Response:**

Thank you for this important clarification request. The parameter ranges were selected based on standard TIG welding guidelines for 304L stainless steel and preliminary pilot experiments. A controlled parameter variation strategy was adopted to generate stable and reproducible defect categories suitable for supervised learning. This explanation has been added.

# Question: What is the source of the normalization values?

**🡺 Response:**

We appreciate this technical question. The normalization mean and standard deviation correspond to the **ImageNet dataset**, ensuring compatibility with the pre-trained ResNet-50 model.

# Question: Add axis titles to Figure 6.

**Response:**
Axis titles (“Weld Class” and “Number of Samples”) have now been added to Figure 6.

# Question: Add references for performance metric equations.

**🡺 Response:**

Thank you for this suggestion. Appropriate references supporting the equations for accuracy, precision, recall, and F1-score have been added.

# Question: Clarify results (training/testing, heatmap, numerical comparisons, conclusion metrics).

**🡺 Response:**

We sincerely appreciate this detailed feedback. The Results section has been carefully revised to:

- Clarify that the reported metrics correspond to the testing phase under five-fold cross-validation,
- Include the referenced heatmap (Figure 8),
- Provide clearer numerical comparisons in the discussion of Figure 11,

Include additional evaluation metrics (precision, recall, F1-score) in both the Results and Conclusion sections.

Once again, we sincerely thank the reviewer for the constructive comments and valuable suggestions. These improvements have significantly enhanced the clarity, technical completeness, and overall quality of the manuscript.

Sincerely,

**All Authors**
