# Supplementary material for: TIG welding defect detection using ResNet and Random Forest
Source: PLoS One. 2026 Jul 30;21(7):e0353276. doi: 10.1371/journal.pone.0353276 (PMC13422830; doi:10.1371/journal.pone.0353276)
Supplement: Attachment — Submitted filename: Response to Reviewers_PONE-D-25-29965__2026_06_02.docx [file pone.0353276.s004.docx]

**Response to Reviewers**

Manuscript: TIG Welding Defect Detection Using ResNet and Random Forest

Journal: PLOS ONE | Manuscript ID: PONE-D-25-29965

We thank the Reviewers for the valuable feedback. The changes made are described below.

Reviewer #1:

The authors have adequately addressed the issues raised; however, they have not included most of this information in the revised manuscript (i.e., the base current, value of the pulse frequency, welding speed, shielding gas flow rate, type and diameter of tungsten electrode, the fact that DCSP was used, chemical composition of the base metal, reference to ISO 5817, etc.).

*Response:*

*All the information has now been added to the Experiment Setup section of the revised manuscript. The following information has been included: welding polarity using DCSP, tungsten electrode type WT20 with a diameter of 1.6 mm, base current of 53 A, pulse frequency ranging from 4.55 to 5.56 Hz, welding speed of 100–105 mm/min, shielding gas flow rate of 19–22 CFH, base metal of 304 stainless steel, and weld quality categories defined in accordance with ISO 5817.*

Reviewer #3:

The abstract needs to be modified. Why CNN architecture is selected in this research, why not SVM with hyperparameter tuning methods? Why RF only, why not NBC? The conclusion may be modified. The computational complexity may be included.

*Response:*

*The selection of CNN over SVM was based on the nature of the input data. CNN-based architectures can learn visual representations directly from raw images without requiring manually designed features, which makes them more suitable for complex weld images. For the choice of Random Forest over NBC, Random Forest does not assume feature independence among input variables, making it a more appropriate choice when working with deep features that tend to be correlated. The experimental results further support this, with the proposed hybrid achieving higher performance than both SVM and standalone Random Forest.* *These considerations are reflected in the following revisions to the manuscript:*

*Abstract: the following sentence has been added: "ResNet50 extracts hierarchical visual representations through deep residual connections, enabling automatic feature learning, while Random Forest performs the final classification with robustness against overfitting on high-dimensional features."*

*Introduction: to address the question of why CNN-based approaches are preferred, the following sentence has been added at the end of the literature review, before the statement of the proposed method: "Unlike approaches relying on manually engineered features, CNN-based architectures combined with ensemble classifiers such as Random Forest can automatically learn visual representations directly from raw images, offering improved scalability and robustness for complex weld defect classification."*

*Conclusion: it has been revised to include a comment on computational efficiency. The following sentence has been added: "The proposed approach combines ResNet50 for deep visual feature extraction and Random Forest for classification on compact feature vectors rather than raw pixel data, achieving high accuracy while maintaining computational efficiency, making it practical for identifying TIG weld quality.”*

Reviewer #4:

There are some similar minor typographical errors throughout the text (such as extra spaces, inconsistent singular and plural forms, etc.). It is recommended that the author use the spelling and grammar checking functions of word processing software to quickly review and further improve the language quality.

*Response:*

*A thorough proofreading of the entire manuscript has been conducted. All identified issues have been corrected, including: singular/plural inconsistencies, grammatical errors (e.g., "data augmentation be done" corrected to "data augmentation is applied", "typical algorithm" corrected to "typical algorithms", "combined Random Forest" corrected to "combined with Random Forest"), and a duplicated word in the text.*

Sincerely yours,
