# Supplementary material for: TIG welding defect detection using ResNet and Random Forest
Source: PLoS One. 2026 Jul 30;21(7):e0353276. doi: 10.1371/journal.pone.0353276 (PMC13422830; doi:10.1371/journal.pone.0353276)
Supplement: Peer review history

# Peer review: Decision Letter 0

**DOI:** 10.1371/journal.pone.0353276.r001

**Authors:** José Galán

3 Oct 2025

PONE-D-25-29965

TIG Welding Detection Using Resnet And Random Forest

PLOS ONE

Dear Dr. Pham,

Thank you for submitting your manuscript to PLOS ONE. After careful consideration, we feel that it has merit but does not fully meet PLOS ONE’s publication criteria as it currently stands. Therefore, we invite you to submit a revised version of the manuscript that addresses the points raised during the review process.

Please submit your revised manuscript by Nov 17 2025 11:59PM. If you will need more time than this to complete your revisions, please reply to this message or contact the journal office at plosone@plos.org. When you're ready to submit your revision, log on to https://www.editorialmanager.com/pone/ and select the 'Submissions Needing Revision' folder to locate your manuscript file.

Please include the following items when submitting your revised manuscript:

- A rebuttal letter that responds to each point raised by the academic editor and reviewer(s). You should upload this letter as a separate file labeled 'Response to Reviewers'.
- A marked-up copy of your manuscript that highlights changes made to the original version. You should upload this as a separate file labeled 'Revised Manuscript with Track Changes'.
- An unmarked version of your revised paper without tracked changes. You should upload this as a separate file labeled 'Manuscript'.

If you would like to make changes to your financial disclosure, please include your updated statement in your cover letter. Guidelines for resubmitting your figure files are available below the reviewer comments at the end of this letter.

If applicable, we recommend that you deposit your laboratory protocols in protocols.io to enhance the reproducibility of your results. Protocols.io assigns your protocol its own identifier (DOI) so that it can be cited independently in the future. For instructions see: https://journals.plos.org/plosone/s/submission-guidelines#loc-laboratory-protocols. Additionally, PLOS ONE offers an option for publishing peer-reviewed Lab Protocol articles, which describe protocols hosted on protocols.io. Read more information on sharing protocols at https://plos.org/protocols?utm_medium=editorial-email&utm_source=authorletters&utm_campaign=protocols.

We look forward to receiving your revised manuscript.

Kind regards,

José Manuel Galán, Ph.D.

Academic Editor

PLOS ONE

Journal Requirements:

When submitting your revision, we need you to address these additional requirements.

1. Please ensure that your manuscript meets PLOS ONE's style requirements, including those for file naming. The PLOS ONE style templates can be found at

https://journals.plos.org/plosone/s/file?id=wjVg/PLOSOne_formatting_sample_main_body.pdf and

https://journals.plos.org/plosone/s/file?id=ba62/PLOSOne_formatting_sample_title_authors_affiliations.pdf

2. Please note that PLOS One has specific guidelines on code sharing for submissions in which author-generated code underpins the findings in the manuscript. In these cases, we expect all author-generated code to be made available without restrictions upon publication of the work. Please review our guidelines at https://journals.plos.org/plosone/s/materials-and-software-sharing#loc-sharing-code and ensure that your code is shared in a way that follows best practice and facilitates reproducibility and reuse.

3. We noticed you have some minor occurrence of overlapping text with the following previous publication(s), which needs to be addressed:

Vision based defects detection for Keyhole TIG welding using deep learning with visual explanation - https://doi.org/10.1016/j.jmapro.2020.05.033

In your revision ensure you cite all your sources (including your own works), and quote or rephrase any duplicated text outside the methods section. Further consideration is dependent on these concerns being addressed.

4. We note that your Data Availability Statement is currently as follows: All relevant data are within the manuscript and its Supporting Information files.

Please confirm at this time whether or not your submission contains all raw data required to replicate the results of your study. Authors must share the “minimal data set” for their submission. PLOS defines the minimal data set to consist of the data required to replicate all study findings reported in the article, as well as related metadata and methods (https://journals.plos.org/plosone/s/data-availability#loc-minimal-data-set-definition).

For example, authors should submit the following data:

- The values behind the means, standard deviations and other measures reported;

- The values used to build graphs;

- The points extracted from images for analysis.

Authors do not need to submit their entire data set if only a portion of the data was used in the reported study.

If your submission does not contain these data, please either upload them as Supporting Information files or deposit them to a stable, public repository and provide us with the relevant URLs, DOIs, or accession numbers. For a list of recommended repositories, please see https://journals.plos.org/plosone/s/recommended-repositories.

If there are ethical or legal restrictions on sharing a de-identified data set, please explain them in detail (e.g., data contain potentially sensitive information, data are owned by a third-party organization, etc.) and who has imposed them (e.g., an ethics committee). Please also provide contact information for a data access committee, ethics committee, or other institutional body to which data requests may be sent. If data are owned by a third party, please indicate how others may request data access.

5. If the reviewer comments include a recommendation to cite specific previously published works, please review and evaluate these publications to determine whether they are relevant and should be cited. There is no requirement to cite these works unless the editor has indicated otherwise.

Additional Editor Comments:

Dear Authors,

Thank you for submitting your manuscript entitled “TIG Welding Detection Using ResNet and Random Forest” to PLOS ONE. Your work has been evaluated by two reviewers, who acknowledge the relevance of the study but have raised substantial concerns regarding methodological clarity, completeness of experimental details, literature review, and the presentation of results.

In light of these comments, the decision is Major Revision. Please carefully address all issues raised by the reviewers in your revised version. A detailed response letter indicating how each point has been handled is required with your resubmission.

We look forward to receiving your revised manuscript.

Sincerely

José Manuel Galán

[Note: HTML markup is below. Please do not edit.]

Reviewers' comments:

Reviewer's Responses to Questions

**Comments to the Author**

1. Is the manuscript technically sound, and do the data support the conclusions?

The manuscript must describe a technically sound piece of scientific research with data that supports the conclusions. Experiments must have been conducted rigorously, with appropriate controls, replication, and sample sizes. The conclusions must be drawn appropriately based on the data presented.

Reviewer #1: Partly

Reviewer #2: Yes

**********

2. Has the statistical analysis been performed appropriately and rigorously?

Reviewer #1: Yes

Reviewer #2: Yes

**********

3. Have the authors made all data underlying the findings in their manuscript fully available?

The PLOS Data policy requires authors to make all data underlying the findings described in their manuscript fully available without restriction, with rare exception (please refer to the Data Availability Statement in the manuscript PDF file). The data should be provided as part of the manuscript or its supporting information, or deposited to a public repository. For example, in addition to summary statistics, the data points behind means, medians and variance measures should be available. If there are restrictions on publicly sharing data—e.g. participant privacy or use of data from a third party—those must be specified.

Reviewer #1: Yes

Reviewer #2: Yes

**********

4. Is the manuscript presented in an intelligible fashion and written in standard English?

PLOS ONE does not copyedit accepted manuscripts, so the language in submitted articles must be clear, correct, and unambiguous. Any typographical or grammatical errors should be corrected at revision, so please note any specific errors here.

Reviewer #1: Yes

Reviewer #2: Yes

**********

5. Review Comments to the Author

Please use the space provided to explain your answers to the questions above. You may also include additional comments for the author, including concerns about dual publication, research ethics, or publication ethics. (Please upload your review as an attachment if it exceeds 20,000 characters)

Reviewer #1: In a quality control process carried out on welded specimens, the conditions and parameters used in the welding process must be clearly specified. This is not the case in the present article, where much information is missing in this respect.

Please consider the following recommendations:

-Are we talking about pulsed or non-pulsed TIG? From what is mentioned in table 1 and in the previous paragraph, it seems that it is pulsed TIG, but if so it should be explicitly stated.

-In Table 1, the welding current is mentioned, which is understood to be the pulse current, but what is the value of the base current?

-In Table 1, pulse time is the pulse current duration? If so, the values shown are excessively high.

-What is the value of the pulse frequency?

-What is the value of the welding speed?

-What is the value of the shielding gas flow rate?

-Is filler material used? If so, please indicate which one.

-Tungsten electrode is pure or alloyed?

-What is the value of the electrode diameter?

-Is direct current in straight polarity or alternating current used in the welding process?

-Please, indicate de chemical composition of the base metal.

-In the text, it is mentioned: “four distinct welding states were identified: lack of fusion, overheating, uneven weld, and normal weld”, please provide some reference to support this classification.

Reviewer #2: 1. The title needs to be modified, I suggest including the term “welding defect detection/classification” instead of “welding detection” to make it more accurate and aligned with the content of the manuscript.

2. The abstract needs to be rewritten and modified for further clarification. It should state the AI methods/algorithms that were used and also the evaluation techniques applied to validate the results.

Also, the final sentence is unclear, the author mentions “this method performs better …”, it is not clear which specific method is being referred to. Please revise to ensure clarity.

3. The references used in the paper are quite outdated. It is recommended to include more recent studies 2024-2025, especially in the related work section to strengthen the contribution and relevance of the paper. Here are suggested publications in the same study field:

- Khan, I. U., Aslam, N., Aboulnour, M., Bashamakh, A., Alghool, F., Alsuwayan, N., Alturaif, R., Gull, H., Iqbal, S. Z., & Hussain, T. (2024). Deep learning-based surface defect detection in steel products using convolutional neural networks. Mathematical Modelling of Engineering Problems, 11, 3006– 3014. https:// doi. org/ 10. 18280/ mmep. 111113

- Palma-Ramírez, D., Ross-Veitía, B. D., Font-Ariosa, P., Espinel-Hernández, A., Sanchez-Roca, A., Carvajal-Fals, H., Nuñez-Alvarez, J. R., & Hernández-Herrera, H. (2024). Deep convolutional neural network for weld defect classification in radiographic images. Heliyon, 10, 1–11. https:// doi. org/ 10. 1016/j. heliy on. 2024. e30590

- Zhang, Q., Zhang, K., Pan, K., & Huang, W. (2024). Image defect classification of surface mount technology welding based on the improved ResNet model. Journal of Engineering Research (Kuwait), 12, 154–162. https://d oi. org/ 10. 1016/j. jer. 2024. 02. 007

- Elhendawy, G.A., El-Taybany, Y. Machine Vision-Assisted Welding Defect Detection System with Convolutional Neural Networks. Int. J. Precis. Eng. Manuf. (2025). https://doi.org/10.1007/s12541-025-01281-y

4. In the introduction section:

- in the first paragraph, the sentence “defects may result in the welds” is too general. It would be more informative to mention specific examples of common weld defects (e.g., porosity, cracks, lack of fusion) and support them with appropriate references.

- The second paragraph, it is recommended to split it into two parts: one describing online monitoring methods and the other describing offline monitoring methods.

- There are several issues with the references and writing style. For example, reference no. 18 is incorrectly cited as 2019, while it is actually a 2021 source. The phrase “and his colleagues” should be replaced by the standard academic style “et al.”.

Furthermore, the literature review section should be written in the past tense, whereas the final part that states the research aim and contribution can be written in the present or present perfect tense. Please revise to ensure tense consistency throughout the manuscript.

5. The methodology section needs some references to support the entire information about ResNet and RF combination benefits. Please cite relevant works that demonstrate or justify this advantage.

6. related to the previous comment, the author compared their proposed combination model with other machine learning methods (e.g., RF, SVM) and CNN architectures (e.g., VGG, ResNet, GoogLeNet/Inception), Please add a short description for each method (in Methodology section) to be more understandable.

7. Is the medical endoscopic camera used sufficient to reliably capture different types of weld defects. Please justify the adequacy of this device for weld defect detection, and if it is possible, please provide supporting references where similar cameras or optical setups have been successfully used before.

8. The experimental parameters listed in Table 1 (welding current, pulse time, and corresponding defect types), it is not clear whether these values were selected based on a specific Design of Experiments (DoE) approach, prior studies, or trial-and-error. Please clarify how choosing these parameter ranges and provide references or justification for this selection.

9. The author states that the input images were normalized using mean values and standard deviations, Please clarify the source of these values.

10. Figure 6 >> add the axis title (weld class & no. of samples).

11. Please add the references you get the equations of the performance metrices.

12. The results section requires further clarification:

- Table 2 only shows the training accuracy. What about the testing and validation results for completeness.

- A heatmap is mentioned in the text, but no corresponding figures are included. Please add the relevant images to support the discussion.

- In the last paragraph of the results (related to Figure 11), the explanation is not clear enough. Provide a more detailed description and include the numerical results to make the findings easier to understand and compare.

- Also, the conclusion should include additional performance evaluation metrics (e.g., precision, recall, F1-score) with numerical values, instead of reporting only accuracy, to give a more comprehensive assessment of the proposed method.

**********

6. PLOS authors have the option to publish the peer review history of their article (what does this mean?). If published, this will include your full peer review and any attached files.

If you choose “no”, your identity will remain anonymous but your review may still be made public.

**Do you want your identity to be public for this peer review?**  For information about this choice, including consent withdrawal, please see our Privacy Policy.

Reviewer #1: No

Reviewer #2: No

**********

[NOTE: If reviewer comments were submitted as an attachment file, they will be attached to this email and accessible via the submission site. Please log into your account, locate the manuscript record, and check for the action link "View Attachments". If this link does not appear, there are no attachment files.]

While revising your submission, please upload your figure files to the Preflight Analysis and Conversion Engine (PACE) digital diagnostic tool, https://pacev2.apexcovantage.com/. PACE helps ensure that figures meet PLOS requirements. To use PACE, you must first register as a user. Registration is free. Then, login and navigate to the UPLOAD tab, where you will find detailed instructions on how to use the tool. If you encounter any issues or have any questions when using PACE, please email PLOS at figures@plos.org. Please note that Supporting Information files do not need this step.

# Author comment: Author response to Decision Letter 1

**DOI:** 10.1371/journal.pone.0353276.r002

1 Apr 2026

Reviewer #1:

Question: Are we talking about pulsed or non-pulsed TIG? It seems to be pulsed TIG, but this should be explicitly stated.

Response:

Thank you very much for this important observation. We sincerely apologize for the lack of clarity in the original version. The welding process used in this study is pulsed TIG welding, and this has now been explicitly stated in the revised manuscript. The pulse parameters (pulse current, pulse ON time, and pulse OFF time) have been clearly specified to ensure full transparency and reproducibility.

Question: In Table 1, the welding current is mentioned. What is the value of the base current?

Response:

We sincerely appreciate the reviewer’s attention to this detail. The peak (pulse) current ranged from 115–125 A, and the base current was maintained at approximately 40% of the peak current. This setting ensured arc stability and controlled heat input during pulsed TIG welding. This information has now been clearly added to the manuscript.

Question: Is the pulse time the pulse current duration? The values appear excessively high. What is the pulse frequency?

Response:

Thank you for this thoughtful comment. Yes, the pulse time refers to the pulse current (ON time) duration. The pulse frequency was calculated using:

f=\frac{1}{t_{ON}+t_{OFF}}\bigm

Pulse ON time: 80–150 ms

Pulse OFF time: 100 ms

Frequency range: 4–5.56 Hz

These values fall within standard pulsed TIG welding practice for stainless steel and have now been clarified in the revised manuscript.

Question: What is the value of the welding speed?

Response:

Thank you for pointing out this missing information. The welding speed was maintained within the range of 100–105 mm/min, and this has now been clearly specified in the revised version.

Question: What is the shielding gas flow rate?

Response:

We appreciate the reviewer’s careful attention to completeness. The shielding gas flow rate ranged from 9–10 L/min, and this information has been added to the manuscript.

Question: Is filler material used?

Response:

Thank you for raising this point. The welding was performed as autogenous welding, meaning no filler material was used. This has now been explicitly clarified.

Question: Is the tungsten electrode pure or alloyed?

Response:

We sincerely thank the reviewer for this question. A WT20 (2% thoriated tungsten) electrode, which is an alloyed type, was used for welding 304L stainless steel. This information has been added to ensure clarity.

Question: What is the electrode diameter?

Response:

The electrode diameter was 1.6 mm (1/16 inch). This specification has now been included in the revised manuscript.

Question: Was DC straight polarity or AC used?

Response:

Thank you for this important clarification request. The process employed Direct Current Straight Polarity (DCSP), which is standard for pulsed TIG welding of 304L stainless steel. This has now been clearly stated.

Question: Please indicate the chemical composition of the base metal.

Response:

We greatly appreciate this suggestion. The base metal was 304L stainless steel, with chemical composition (according to ASTM/EN standards) as follows:

C: ≤ 0.030%

Cr: 17.5–19.5%

Ni: 8.0–12.0%

Mn: ≤ 2.0%

Si: ≤ 1.0%

Fe: Balance

These details have been added to the manuscript.

Question: Please provide references supporting the classification of weld states.

Response:

Thank you for this valuable recommendation. The classification of weld states (lack of fusion, overheating, uneven weld, normal weld) has been clarified and supported with reference to ISO 5817, which provides standardized weld quality assessment criteria. The appropriate citation has been included in the revised manuscript.

Reviewer #2:

Question: The title should include “welding defect detection/classification.”

Response:

We sincerely thank the reviewer for this helpful suggestion. The title has been revised to:

“TIG Welding Defect Detection Using ResNet and Random Forest.”

Question: The abstract needs clarification of AI methods and evaluation strategy.

Response:

We truly appreciate this constructive comment. The abstract has been completely revised to clearly describe:

The applied AI methods (ResNet-50 and Random Forest),

The evaluation strategy (five-fold cross-validation),

The performance metrics (accuracy, precision, recall, F1-score),

And explicitly clarify that the proposed hybrid ResNet–Random Forest model achieved superior performance compared with standalone approaches.

Question: The references are outdated. Please include recent studies (2024–2025).

Response:

Thank you very much for this important recommendation. The Related Work section has been updated to include recent publications from 2024–2025, including the suggested references. This significantly strengthens the relevance and timeliness of our work.

________________________________________

Question: Improvements needed in the Introduction (defect examples, structure, tense consistency, citation corrections).

Response:

We sincerely appreciate this detailed feedback. The Introduction has been carefully revised to:

Include specific examples of common weld defects (porosity, cracks, lack of fusion),

Clearly separate online and offline monitoring methods,

Correct citation inaccuracies,

Ensure consistent academic writing style and tense usage throughout.

Question: Please justify the ResNet + Random Forest combination with references.

Response:

Thank you for this insightful suggestion. Additional references have been incorporated into the Methodology section to support the advantages of combining deep feature extraction (ResNet) with traditional machine learning classifiers (Random Forest), particularly in terms of generalization and robustness.

Question: Please add short descriptions of the comparative models.

Response:

We appreciate this helpful comment. Concise descriptions of VGG, ResNet, SVM, Random Forest, and Inception models have now been included in the Methodology section for better clarity and completeness.

Question: Is the endoscopic camera sufficient for weld defect detection?

Response:

We sincerely thank the reviewer for this thoughtful question. The selected endoscopic camera (640 × 480 resolution, 15 mm focus distance) provides a spatial resolution of approximately 0.03–0.05 mm per pixel, which is sufficient to detect millimeter-scale weld defects within pipe geometry. Stable mounting and controlled illumination ensured consistent and reliable image acquisition. This justification has been added to the manuscript.

Question: How were welding parameter ranges selected?

Response:

Thank you for this important clarification request. The parameter ranges were selected based on standard TIG welding guidelines for 304L stainless steel and preliminary pilot experiments. A controlled parameter variation strategy was adopted to generate stable and reproducible defect categories suitable for supervised learning. This explanation has been added.

Question: What is the source of the normalization values?

Response:

We appreciate this technical question. The normalization mean and standard deviation correspond to the ImageNet dataset, ensuring compatibility with the pre-trained ResNet-50 model.

Question: Add axis titles to Figure 6.

Response:

Axis titles (“Weld Class” and “Number of Samples”) have now been added to Figure 6.

Question: Add references for performance metric equations.

Response:

Thank you for this suggestion. Appropriate references supporting the equations for accuracy, precision, recall, and F1-score have been added.

Question: Clarify results (training/testing, heatmap, numerical comparisons, conclusion metrics).

Response:

We sincerely appreciate this detailed feedback. The Results section has been carefully revised to:

Clarify that the reported metrics correspond to the testing phase under five-fold cross-validation,

Include the referenced heatmap (Figure 8),

Provide clearer numerical comparisons in the discussion of Figure 11,

Include additional evaluation metrics (precision, recall, F1-score) in both the Results and Conclusion sections.

Attachment Submitted filename: Response to Reviewers 2_PONE-D-25-29965_1.docx

# Peer review: Decision Letter 1

**DOI:** 10.1371/journal.pone.0353276.r003

**Authors:** José Galán, José Galán

25 May 2026

PONE-D-25-29965R1

TIG welding defect detection using Resnet and Random forest

PLOS One

Dear Dr. Son Minh,

Thank you for submitting your manuscript to PLOS ONE. After careful consideration, we feel that it has merit but does not fully meet PLOS ONE’s publication criteria as it currently stands. Therefore, we invite you to submit a revised version of the manuscript that addresses the points raised during the review process.

Please submit your revised manuscript by Jul 09 2026 11:59PM. If you will need more time than this to complete your revisions, please reply to this message or contact the journal office at plosone@plos.org. When you're ready to submit your revision, log on to https://www.editorialmanager.com/pone/ and select the 'Submissions Needing Revision' folder to locate your manuscript file.

Please include the following items when submitting your revised manuscript:

- A letter that responds to each point raised by the academic editor and reviewer(s). You should upload this letter as a separate file labeled 'Response to Reviewers'.
- A marked-up copy of your manuscript that highlights changes made to the original version. You should upload this as a separate file labeled 'Revised Manuscript with Track Changes'.
- An unmarked version of your revised paper without tracked changes. You should upload this as a separate file labeled 'Manuscript'.

If you would like to make changes to your financial disclosure, please include your updated statement in your cover letter. Guidelines for resubmitting your figure files are available below the reviewer comments at the end of this letter.

If applicable, we recommend that you deposit your laboratory protocols in protocols.io to enhance the reproducibility of your results. Protocols.io assigns your protocol its own identifier (DOI) so that it can be cited independently in the future. For instructions see: https://journals.plos.org/plosone/s/submission-guidelines#loc-laboratory-protocols. Additionally, PLOS ONE offers an option for publishing peer-reviewed Lab Protocol articles, which describe protocols hosted on protocols.io. Read more information on sharing protocols at https://plos.org/protocols?utm_medium=editorial-email&utm_source=authorletters&utm_campaign=protocols.

As the corresponding author, your ORCID iD is verified in the submission system and will appear in the published article. PLOS supports the use of ORCID, and we encourage all coauthors to register for an ORCID iD and use it as well. Please encourage your coauthors to verify their ORCID iD within the submission system before final acceptance, as unverified ORCID iDs will not appear in the published article. *Only*  the individual author can complete the verification step; PLOS staff *cannot*  verify ORCID iDs on behalf of authors.

We look forward to receiving your revised manuscript.

Kind regards,

José Manuel Galán, Ph.D.

Academic Editor

PLOS One

Journal Requirements:

If the reviewer comments include a recommendation to cite specific previously published works, please review and evaluate these publications to determine whether they are relevant and should be cited. There is no requirement to cite these works unless the editor has indicated otherwise.

Please review your reference list to ensure that it is complete and correct. If you have cited papers that have been retracted, please include the rationale for doing so in the manuscript text, or remove these references and replace them with relevant current references. Any changes to the reference list should be mentioned in the rebuttal letter that accompanies your revised manuscript. If you need to cite a retracted article, indicate the article’s retracted status in the References list and also include a citation and full reference for the retraction notice.

Additional Editor Comments:

Dear Authors,

Thank you for submitting the revised version of your manuscript, “TIG welding defect detection using Resnet and Random forest”, to PLOS ONE.

The reviewers acknowledge that the manuscript has improved and that most previous concerns have been addressed. However, a final minor revision is required before the manuscript can be considered for acceptance.

Please ensure that all relevant technical details provided in your response letter are also clearly included in the manuscript, particularly the welding parameters, electrode specifications, polarity, base metal composition, and reference to the applicable weld classification standard.

Please also revise the abstract and conclusion to clarify the rationale for using the selected ResNet/CNN and Random Forest approach, briefly discuss why alternative classifiers were not used or were less suitable, and add a short comment on computational complexity.

Finally, please conduct a careful proofreading to correct minor typographical, grammatical, spacing, and consistency issues.

No new experiments appear to be required. Please submit a revised manuscript and a concise response letter indicating where the changes have been made.

Sincerely,

José M. Galán

[Note: HTML markup is below. Please do not edit.]

Reviewers' comments:

Reviewer's Responses to Questions

**Comments to the Author**

1. If the authors have adequately addressed your comments raised in a previous round of review and you feel that this manuscript is now acceptable for publication, you may indicate that here to bypass the “Comments to the Author” section, enter your conflict of interest statement in the “Confidential to Editor” section, and submit your "Accept" recommendation.

Reviewer #1: (No Response)

Reviewer #3: All comments have been addressed

Reviewer #4: All comments have been addressed

**********

2. Is the manuscript technically sound, and do the data support the conclusions?

The manuscript must describe a technically sound piece of scientific research with data that supports the conclusions. Experiments must have been conducted rigorously, with appropriate controls, replication, and sample sizes. The conclusions must be drawn appropriately based on the data presented.

Reviewer #1: Partly

Reviewer #3: Yes

Reviewer #4: Yes

**********

3. Has the statistical analysis been performed appropriately and rigorously?

Reviewer #1: Yes

Reviewer #3: No

Reviewer #4: Yes

**********

4. Have the authors made all data underlying the findings in their manuscript fully available?

The PLOS Data policy requires authors to make all data underlying the findings described in their manuscript fully available without restriction, with rare exception (please refer to the Data Availability Statement in the manuscript PDF file). The data should be provided as part of the manuscript or its supporting information, or deposited to a public repository. For example, in addition to summary statistics, the data points behind means, medians and variance measures should be available. If there are restrictions on publicly sharing data—e.g. participant privacy or use of data from a third party—those must be specified.

Reviewer #1: Yes

Reviewer #3: Yes

Reviewer #4: Yes

**********

5. Is the manuscript presented in an intelligible fashion and written in standard English?

PLOS ONE does not copyedit accepted manuscripts, so the language in submitted articles must be clear, correct, and unambiguous. Any typographical or grammatical errors should be corrected at revision, so please note any specific errors here.

Reviewer #1: Yes

Reviewer #3: Yes

Reviewer #4: Yes

**********

6. Review Comments to the Author

Please use the space provided to explain your answers to the questions above. You may also include additional comments for the author, including concerns about dual publication, research ethics, or publication ethics. (Please upload your review as an attachment if it exceeds 20,000 characters)

Reviewer #1: In their response to the reviewers, the authors have adequately addressed the issues raised by this reviewer; however, they have not included most of this information, even though the authors explicitly state that they do include this data, in the revised manuscript (i.e. the base current, value of the pulse frequency, the value of the welding speed, the value of the shielding gas flow rate, the type and diameter Tungsten electrode, the fact that the welding process used Direct Current Straight Polarity (DCSP), the chemical composition of the base metal, the reference to ISO 5817, etc.). I think this information is useful and should be included in the revised manuscript.

Reviewer #3: The abstract needs to be modified. why CNN architecture are selected in this research why not SVM with hyperparameter tuning methods? Why RF only why not NBC? The conclusion may be modified. The cocmputational complexity may be included.

Reviewer #4: Reviewer # 1 Comment (11 in total):

All responses have been fully received, and relevant information has been added to the main text, including pulse parameters, base current, welding speed, protective gas flow rate, tungsten electrode type/diameter, polarity, base metal composition, and reference to weld classification standards.

Reviewer # 2 comments (12 in total):

The title, abstract, introduction, references, model description, camera resolution explanation, parameter selection basis, normalization source, Figure 6 axis title, formula citation, result clarification, etc. have all been modified as required.

Review opinion: Suggest a global inspection

There are some similar minor typographical errors throughout the text (such as extra spaces, inconsistent singular and plural forms, etc.). It is recommended that the author use the spelling and grammar checking functions of word processing software to quickly review and further improve the language quality.

**********

7. PLOS authors have the option to publish the peer review history of their article (what does this mean?). If published, this will include your full peer review and any attached files.

If you choose “no”, your identity will remain anonymous but your review may still be made public.

**Do you want your identity to be public for this peer review?**  For information about this choice, including consent withdrawal, please see our Privacy Policy.

Reviewer #1: No

Reviewer #3: **Yes:**  Harikumar Rajaguru

Reviewer #4: No

**********

[NOTE: If reviewer comments were submitted as an attachment file, they will be attached to this email and accessible via the submission site. Please log into your account, locate the manuscript record, and check for the action link "View Attachments". If this link does not appear, there are no attachment files.]

To ensure your figures meet our technical requirements, please review our figure guidelines: https://journals.plos.org/plosone/s/figures

You may also use PLOS’s free figure tool, NAAS, to help you prepare publication quality figures: https://journals.plos.org/plosone/s/figures#loc-tools-for-figure-preparation.

NAAS will assess whether your figures meet our technical requirements by comparing each figure against our figure specifications.

# Author comment: Author response to Decision Letter 2

**DOI:** 10.1371/journal.pone.0353276.r004

19 Jun 2026

Reviewer #1:

The authors have adequately addressed the issues raised; however, they have not included most of this information in the revised manuscript (i.e., the base current, value of the pulse frequency, welding speed, shielding gas flow rate, type and diameter of tungsten electrode, the fact that DCSP was used, chemical composition of the base metal, reference to ISO 5817, etc.).

Response:

All the information has now been added to the Experiment Setup section of the revised manuscript. The following information has been included: welding polarity using DCSP, tungsten electrode type WT20 with a diameter of 1.6 mm, base current of 53 A, pulse frequency ranging from 4.55 to 5.56 Hz, welding speed of 100–105 mm/min, shielding gas flow rate of 19–22 CFH, base metal of 304 stainless steel, and weld quality categories defined in accordance with ISO 5817.

Reviewer #3:

The abstract needs to be modified. Why CNN architecture is selected in this research, why not SVM with hyperparameter tuning methods? Why RF only, why not NBC? The conclusion may be modified. The computational complexity may be included.

Response:

The selection of CNN over SVM was based on the nature of the input data. CNN-based architectures can learn visual representations directly from raw images without requiring manually designed features, which makes them more suitable for complex weld images. For the choice of Random Forest over NBC, Random Forest does not assume feature independence among input variables, making it a more appropriate choice when working with deep features that tend to be correlated. The experimental results further support this, with the proposed hybrid achieving higher performance than both SVM and standalone Random Forest. These considerations are reflected in the following revisions to the manuscript:

Abstract: the following sentence has been added: "ResNet50 extracts hierarchical visual representations through deep residual connections, enabling automatic feature learning, while Random Forest performs the final classification with robustness against overfitting on high-dimensional features."

Introduction: to address the question of why CNN-based approaches are preferred, the following sentence has been added at the end of the literature review, before the statement of the proposed method: "Unlike approaches relying on manually engineered features, CNN-based architectures combined with ensemble classifiers such as Random Forest can automatically learn visual representations directly from raw images, offering improved scalability and robustness for complex weld defect classification."

Conclusion: it has been revised to include a comment on computational efficiency. The following sentence has been added: "The proposed approach combines ResNet50 for deep visual feature extraction and Random Forest for classification on compact feature vectors rather than raw pixel data, achieving high accuracy while maintaining computational efficiency, making it practical for identifying TIG weld quality.”

Reviewer #4:

There are some similar minor typographical errors throughout the text (such as extra spaces, inconsistent singular and plural forms, etc.). It is recommended that the author use the spelling and grammar checking functions of word processing software to quickly review and further improve the language quality.

Response:

A thorough proofreading of the entire manuscript has been conducted. All identified issues have been corrected, including: singular/plural inconsistencies, grammatical errors (e.g., "data augmentation be done" corrected to "data augmentation is applied", "typical algorithm" corrected to "typical algorithms", "combined Random Forest" corrected to "combined with Random Forest"), and a duplicated word in the text.

Attachment Submitted filename: Response to Reviewers_PONE-D-25-29965__2026_06_02.docx

# Editor report: Decision Letter 2

**DOI:** 10.1371/journal.pone.0353276.r005

**Authors:** José Galán, José Galán, José Galán

22 Jun 2026

TIG welding defect detection using Resnet and Random forest

PONE-D-25-29965R2

Dear Dr. Son Minh,

We’re pleased to inform you that your manuscript has been judged scientifically suitable for publication and will be formally accepted for publication once it meets all outstanding technical requirements.

Within one week, you’ll receive an e-mail detailing the required amendments. When these have been addressed, you’ll receive a formal acceptance letter and your manuscript will be scheduled for publication.

An invoice will be generated when your article is formally accepted. Please note, if your institution has a publishing partnership with PLOS and your article meets the relevant criteria, all or part of your publication costs will be covered. Please make sure your user information is up-to-date by logging into Editorial Manager at Editorial Manager® and clicking the ‘Update My Information' link at the top of the page. For questions related to billing, please contact billing support.

If your institution or institutions have a press office, please notify them about your upcoming paper to help maximize its impact. If they’ll be preparing press materials, please inform our press team as soon as possible -- no later than 48 hours after receiving the formal acceptance. Your manuscript will remain under strict press embargo until 2 pm Eastern Time on the date of publication. For more information, please contact onepress@plos.org.

Kind regards,

José Manuel Galán, Ph.D.

Academic Editor

PLOS One

Additional Editor Comments (optional):

Reviewers' comments:

# Editor report: Acceptance letter

**DOI:** 10.1371/journal.pone.0353276.r006

**Authors:** José Galán, José Galán, José Galán

PONE-D-25-29965R2

PLOS One

Dear Dr. Son Minh,

I'm pleased to inform you that your manuscript has been deemed suitable for publication in PLOS One. Congratulations! Your manuscript is now being handed over to our production team.

At this stage, our production department will prepare your paper for publication. This includes ensuring the following:

* All references, tables, and figures are properly cited

* All relevant supporting information is included in the manuscript submission,

* There are no issues that prevent the paper from being properly typeset

You will receive further instructions from the production team, including instructions on how to review your proof when it is ready. Please keep in mind that we are working through a large volume of accepted articles, so please give us a few days to review your paper and let you know the next and final steps.

Lastly, if your institution or institutions have a press office, please let them know about your upcoming paper now to help maximize its impact. If they'll be preparing press materials, please inform our press team within the next 48 hours. Your manuscript will remain under strict press embargo until 2 pm Eastern Time on the date of publication. For more information, please contact onepress@plos.org.

You will receive an invoice from PLOS for your publication fee after your manuscript has reached the completed accept phase. If you receive an email requesting payment before acceptance or for any other service, this may be a phishing scheme. Learn how to identify phishing emails and protect your accounts at https://explore.plos.org/phishing.

If we can help with anything else, please email us at customercare@plos.org.

Thank you for submitting your work to PLOS ONE and supporting open access.

Kind regards,

PLOS ONE Editorial Office Staff

on behalf of

Dr. José Manuel Galán

Academic Editor

PLOS One
